# Changes in epinephrine dispensings and allergy hospitalisations in Sweden in the years following the removal of autoinjector co-payments

**DOI:** 10.3389/falgy.2024.1434461

**Published:** 2024-08-16

**Authors:** Staffan Ahlstedt, Anna Bergström, Lennart Nilsson, Juho E Kivistö, Jennifer L P Protudjer

**Affiliations:** ^1^ Institute of Environmental Medicine, Karolinska Institutet, Stockholm, Sweden; ^2^ Centre of Occupational and Environmental Medicine, Stockholm, Sweden; ^3^ Department of Paediatrics Jönköping, Jönköping, Sweden; ^4^ Department of Biomedical and Clinical Sciences, Linköping University, Linköping, Sweden; ^5^ Allergy Centre, Tampere University Hospital, Tampere, Finland; ^6^ Faculty of Medicine and Health Technology, Tampere University, Tampere, Finland; ^7^ Department of Pediatrics and Child Health, Max Rady College of Medicine, Rady Faculty of Health Sciences, University of Manitoba, Winnipeg, MB, Canada; ^8^ Children’s Hospital Research Institute of Manitoba, Winnipeg, MB, Canada; ^9^ Department of Food and Human Nutritional Sciences, Faculty of Agricultural and Food Sciences, University of Manitoba, Winnipeg, MB, Canada; ^10^ George and Fay Yee Centre for Healthcare Innovation, Winnipeg, MB, Canada

**Keywords:** allergy, anaphylaxis, epidemiology, epinephrine autoinjectors, healthcare utilisation, hospitalisations, pediatrics

## Abstract

**Introduction:**

To understand any possible healthcare system benefits and changes of behavior for the patients with the change in prescription co-payment in Sweden we aimed to provide an update on the trends of EAI dispensings and hospitalizations for the Swedish paediatric population (ages 0–19 years), from 2018 to 2022, including by sex and geographic region.

**Methods:**

Using publically-available, population-level aggregate data from Sweden's National Board of Health and Welfare, we extracted information on annual epinephrine (ATC C01CA24) dispensings per 1,000 inhabitants from 2018 to 2023, overall, as well as stratified by sex, age groups and geographic region; and on inpatient stays 2018–2022 (ICD-10 code T78), anaphylaxis and other allergic reactions, per 100,000 individuals. We compared these estimates to those for adults ages 18 + years, for whom prescription co-payments remained in place.

**Results:**

EAI dispensings remained stable for children and adults across the study period, with the exception of statistically significant decreases amongst dispensings for children across all ages in 2021 (6.65/1,000) and 2022 (7.37/1,000), compared to 2018 (8.63/1,000) (each year *p* = 0.03 compared to 2018 dispensings). National EAI dispensings did not statistically significantly differ from 2018 (8.63/1,000) to 2023 (6.70/1,000) amongst children. EAI dispensings for children ages 5 + years consistently exceed dispensings for adults per 1,000 inhabitants; only children aged 0–4 years had proportionately fewer dispensings. Children ages 0–4 years tended to be hospitalised more often than older children, albeit these differences were not statistically significant (all *p* > 0.97).

**Conclusion:**

Subsequent to the removal of out-of-pocket costs for EAI, dispensings did not increase for children, although more EAI were dispensed to children from age 5 years, compared to younger children. Allergy-related hospitalisations were highest amongst children ages 0–4, lower amongst children ages 5–14 years, and again higher amongst those ages 15–19 years.

## Introduction

Epinephrine autoinjectors [EAI] remain first-line emergency medication in the event of anaphylaxis (1). In some jurisdictions, the copayment costs associated with the purchase of an EAI twin pack range from $113 to $730 American dollars [USD] (2), thus potentially rendering this essential medication out of the financial reach of a substantial number of households in some countries. EAI-related costs in emergency department settings are also substantial. Indeed, in a 2015 study of commonly used drugs in emergency departments, EAI were identified as having the greatest average wholesale price per patient, a calculation based on average wholesale price divided by usage (3). In Sweden, the copayment costs for nearly all pediatric medications, including EAI, were eliminated on 1 January 2016, for children with a Swedish personal identification number (4).

Using population data extracted from national health registers, we previously reported that EAI dispensings tended to increase in the two years (ie., from 2016 to 2017) following the removal of copayments, compared to the years 2006–2015 when modest fees (approximately corresponding to $20 USD per device) (5). Over the same period, annual inpatient stays for anaphylaxis and other allergic reactions, showed an inverse pattern: hospitalisations decreased subsequent to the removal of copayments (5). While causality cannot be interpreted based on these observations, the findings nonetheless provide empirical evidence that greater access to EAI is associated with decreased hospitalisations for allergy-related reasons. To assess if the temporal trend of increased dispensings and decreased hospitalisations, in the present study, we aimed to provide an update on the trends of EAI dispensings and hospitalizations for the Swedish paediatric population (ages 0–19 years), from 2018 to 2023 (EAI dispensings) and from 2018 to 2022 (hospitalisations) and, including by sex and geographic region.

## Methods

This study made use of publically available data from the open, searchable statistical database, namely Sweden's National Board of Health and Welfare, available at https://www.socialstyrelsen.se/statistik-och-data/statistik/statistikdatabasen/ (in Swedish) (6). This database includes aggregate data from the Swedish Prescribed Drug Register. This register, incepted in 2006, is a repository that includes nationwide data on all prescribed drugs that were dispensed from Swedish pharmacies to those with person numbers (in Swedish: personnummer) (7). A person number is unique to each permanent resident and Swedish citizen, and is necessary to access healthcare and most, if not all, other social services. Specifically, we extracted data for annual epinephrine (ATC C01CA24) dispensings from 2018 to 2023, overall, as well as stratified by sex (male, female), age groups (0–4; 5–9; 10–14; and, 15–19 years), and geographic region (nationwide; county). Results for this data extraction are presented as dispensed prescriptions per 1,000 inhabitants, as well as the number of patients to whom EAI were dispensed annually per 1,000 individuals. We also made use of data on diagnoses made in inpatient stays and specialised outpatient care from 2018 to 2022 for ICD-10 code T78, for adverse reactions, not elsewhere classified. These results are presented as annual hospitalizations per 100,000 individuals. As such, it is possible for a given patient to contribute to more than one occasion of EAI dispensings or hospitalisations. To further enhance the interpretation of the impact of no prescription co-payments for children, we compared these results to those for adults, for who modest co-payments remain in place.

Geographic regions were categorised as: *national* (all counties collectively), *large rural areas* encompassing six of Sweden’s 21 counties (8), *other rural areas* encompassing neither large rural areas or the three most populated counties, and *the three most populated counties* in which the three largest cities, namely Stockholm, Gothenburg and Malmo, are located (9).

We calculated changes over time as percent changes (reported as increases or decreases, where appropriate). Statistical significance was set at *p* < 0.05, and calculated based on t-tests between different years; between age groups; between the sexes within a specific age group; and between counties.

To enhance interpretation of the findings, we extracted corresponding data for adults (from age 20 to 85 + years), for whom copayments for medication (including EAI) remain in place. Further, we considered possible differences in proportions between groups (Stata 17.0, College Station, TX), with *p* < 0.05 considered statistically significantly different. As these data were publically available from a database maintained by Swedish authorities, informed consent was neither necessary nor possible to obtain.

## Results

### EAI dispensings

**Figure 1:**
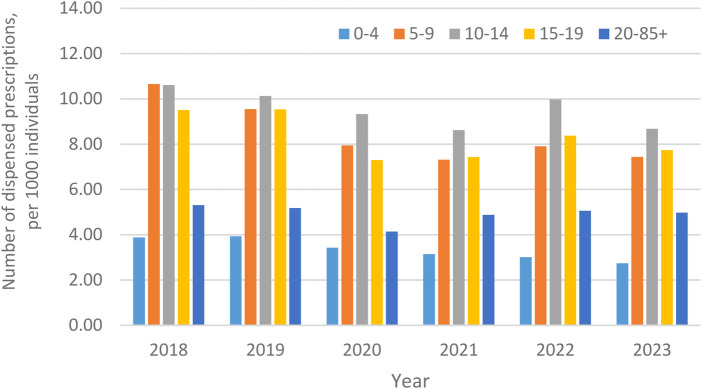
Number of annual epinephrine autoinjector dispensings per 1,000 individuals, from 2018 to 2023, in Sweden, stratified by children (including by age group) and adults (ages 20-85 + years).

The number of annual EAI dispensings per 1,000 inhabitants in children and adults from 2018 to 2023 are shown in Figure 1. Annual EAI dispensings for children ages 5 + years was higher than dispensings for adults, while children aged 0–4 years had proportionately fewer dispensings. With consideration to the number of patients to whom EAI were dispensed annually per 1,000 individuals, a similar pattern amongst children, including by age group, and adults persisted (Supplementary Figure S1).

Amongst children, annual EAI dispensings in 2018 were 8.63/1,000, including 9.87/1,000 for males and 7.31/1,000 for females. These numbers remained relatively stable in 2019 and 2020 (corresponding *p*-values compared to 2018 dispensings: 2019: *p* = 0.69; 2020: *p* = 0.70). Statististically significant decreases were noted in 2021, at 6.65/1,000 overall, and 2022, at 7.37/1,000 overall (each year *p* = 0.03 compared to 2018 dispensings), but failed to reach statistical significance in 2023, at 7.37/1,000 overall (*p* = 0.18 vs. 2018). From 2021 to 2023, decreases in annual EAI dispensings were seen across both sexes. For example, in 2021, dispensings were 7.72/1,000 for males and 5.53 for females, representing decreases of 27.8% and 32.2%, respectively, compared to 2018 dispensings. In comparison, dispensings amongst adults aged 20–85+, remained stable from 2018 (5.31/1,000) to 2023 (4.98/1,000).

**Figure 2:**
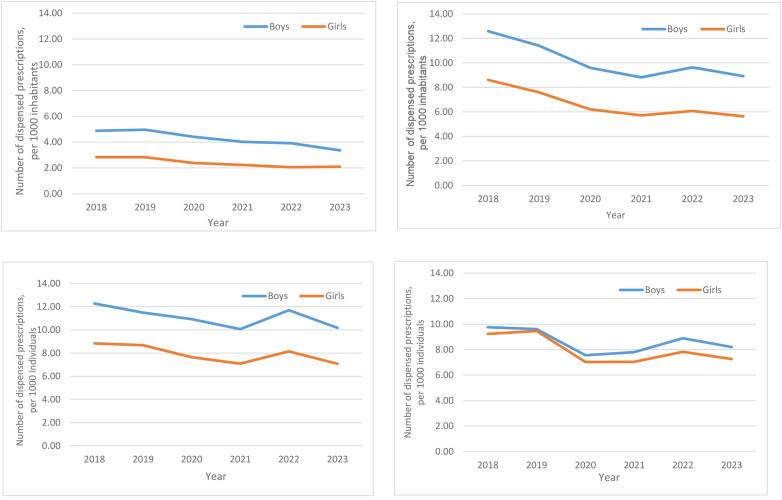
Number of annual epinephrine autoinjector dispensings to paediatric patients, ages 0–19 years, per 1,000 individuals, in Sweden from 2018 to 2023, by sex-stratified age groups. **(A)** 0–4 years, **(B)** 5–9 years, **(C)** 10–14 years, **(D)** 15–19 years.

With consideration to age, annual EAI dispensings were lowest amongst children aged 0–4 years (e.g., 2018: 3.88/1,000), increased significantly at ages 5–9 years (e.g., 2018: 10.66/1,000) and 10–14 years (e.g., 2018: 10.61/1,000), and modestly decreased at ages 15–19 years (e.g., 2018: 9.51/1,000); all *p* < 0.01 vs. ages 0–4 years. Across all years, annual EAI dispensings were non-statistically significantly higher amongst boys than girls at ages 0–4 years, 5–9 years, and 10–14 years (all *p* > 0.05). At ages 15–19 years, annual EAI dispensings tracked very similarly between the sexes (Figure 2).

With consideration to geographic region, the national rates of annual EAI dispensings remained stable from 2018 (8.63/1,000) to 2023 (6.70/1,000) amongst children (Supplementary Table S1). Inter-county variation were substantial but not statistically significantly different, ranging from, 4.34/1,000 to 3.07/1,000, in 2018 and 2023, respectively in to 12.46/1,000 and 10.07/1,000, in 2018 and 2023 respectively, in Stockholm County. Of note, Jämtland County, has a population of 132,466 individuals in the middle of the country with a population density of 2.7 people/km^2^ (10). In contrast, Stockholm County, in which the capital city of Sweden is located, is home to 2,455,914 individuals and has a population density of 377/km^2^ (10). Similar trends persisted throughout the six years (from 2018 to 2023) for which data are reported herein. In each year between 2018 and 2023, annual EAI dispensings per 1,000 nationally were significantly greater for children than adults; this observation was driven almost exclusively by Stockholm county.

### Inpatient stays for anaphylaxis and other allergic reactions

Amongst all age groups combined, from 2018 to 2022, inpatient stays non-significantly increased by 67.9%, from 8.1/100,000 (2018) to 13.6/100,000 (2022; *p* = 0.70 vs. 2018). Amongst children, in 2021, inpatient stays non- significantly decreased to 9.4/100,000 (*p* = 0.99 vs. 2018), a figure which rose, in 2022, to 17.5/100,000 (*p* = 0.93 vs. 2018). Amongst adults ages 29–85+, inpatient stays for anaphylaxis and other allergic reactions amongst adults ages 20–85 + were relatively stable from 2018 to 2021, at 16.9/100,000, and 14.3/100,000, respectively.

**Figure 3:**
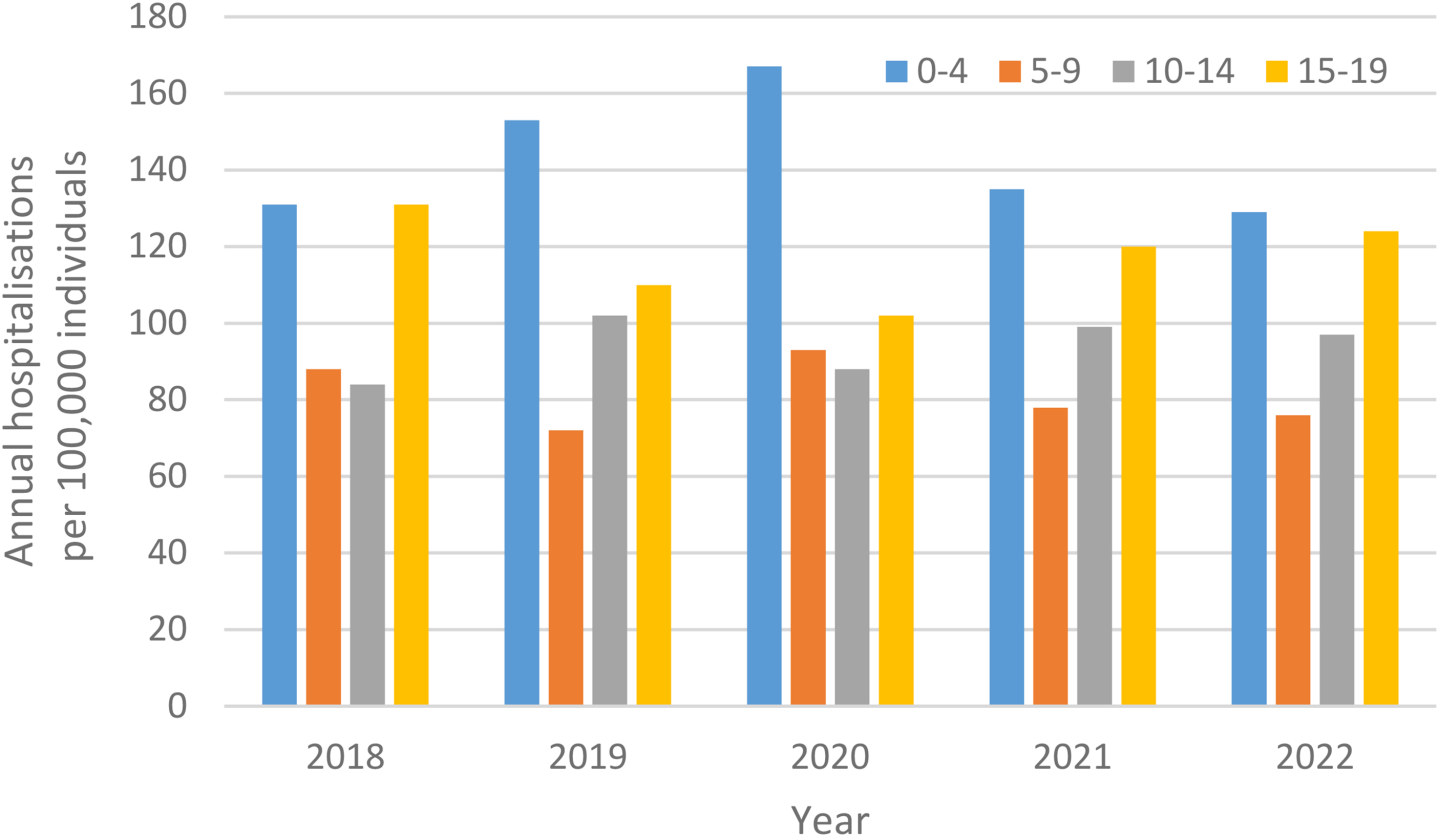
Annual hospitalisations for adverse reactions, not elsewhere classified per 100,000 individuals, from 2018 to 2022, in Sweden, stratified by children (including by age group) and adults.

Hospitalisations per 100,000 individuals for adverse reactions (ICD-10 T78) were highest amongst children ages 0–4 years, then non-significantly decreased amongst those ages 5–9 years, and 10–14 years (all *p* > 0.97; Figure 3). For example, these decreases corresponded to 47.3% and 53.8%, respectively, in 2018. Patterns for those ages 15–19 years were less consistent, with comparable inpatient stays to those noted in the youngest children, in 2018, but which were consistent with inpatient stays for those noted in children ages 5–9 years, and ages 10–14 years in both 2019 and 2020, but which again increased in 2021 to levels similar to those amongst the youngest children.

**Figure 4:**
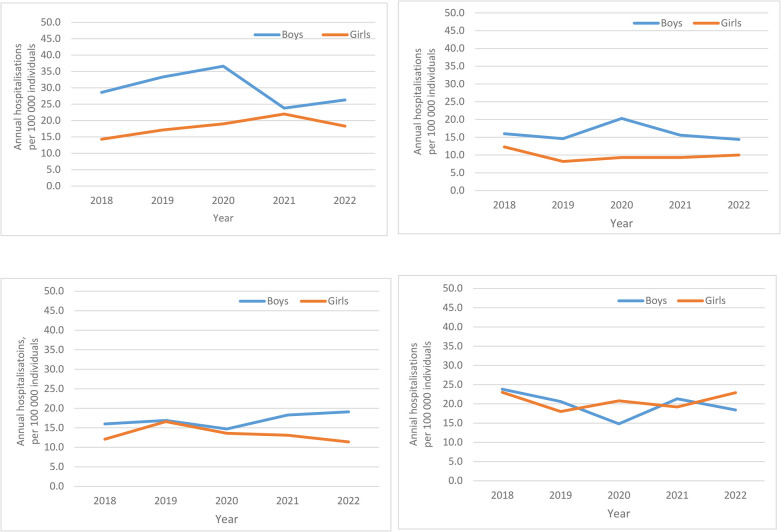
Annual hospitalisations for adverse reactions, not elsewhere classified per 100,000 individuals, ages 0–19 years, in Sweden from 2018- to 2022, by sex-stratified age groups. **(A)** 0–4 years, **(B)** 5–9 years, **(C)** 10–14 years, **(D)** 15–19 years.

With consideration to sex and age, inpatient stays for all years tended to be highest amongst children aged 0–4 years, albeit with notable differences by sex (Figure 4). At ages 0–4 years, 5–9 years, and 10–14 years, the non-statistically significant male to female predominance of hospitalisations was approximately 2:1 (e.g., hospitalisations in 2018 for ages 0–4 years: 28.6/100,000 vs. 14.3/100,000, respectively). In contrast, by ages 15–19 years, this sex difference was attenuated in 2018 and 2019. By, 2020, amongst those ages 15–19 years, inpatient stays had shifted to a female predominance (14.8/100,000 males vs. 20.8/100,000 females, respectively, with a corresponding ratio of 0.71:1.0). In 2021 and 2022, this sex difference was again attenuated.

## Discussion

In the present study, we provide national-level evidence on EAI dispensings and allergy-related hospitalisations from 2018 to 2023, in Sweden, for children, with consideration to age groups and sex, and geographic region. Comparisons were further made to adult EAI dispensings and allergy-related hospitalisations. Children from ages 5–19 years had consistently higher dispensings than children ages 0–4 years, as well as compared to adults. Amongst children, more EAI were dispensed for males than females, although the difference was not statistically significant for each year of the study period. At a national level, EAI dispensings remained stable across the study period, albeit with important inter-county differences. The lowest dispensings per capita were noted in some of the large rural areas, while the highest dispensing rates were noted in the Stockholm county, the most populous county in Sweden, and where the national capital is located. Likewise, in Stockholm county, more EAI were dispensed for children than adults, at approximately 2:1, across the study period. This difference contributed to a statistically greater number of EAI dispensings nationally for children than adults. Indeed, no differences in EAI dispensings between children and adults were found for any county other than Stockholm county. Allergy-related hospitalisations were highest amongst children ages 0–4, lower amongst children in the age groups 5–9 and 10–14 years across all years of the study period. Compared to children ages 0–4 years, amongst children 15–19 years had comparable hospitalisations in 2018, then were notably lower in 2019 and 2020, and then rebounded in 2021 and 2022, to levels comparable to the youngest age group.

In 2016, the Swedish government eliminated out-of-pocket expenses for most prescribed medications, including EAI for children (4). In the two years immediately thereafter (ie. 2016–2017), EAI dispensings increased and allergy-related hospitalisations decreased, compared to the years immediately prior to the removal of costs. Interestingly, we demonstrated that annual EAI dispensings to both children and adults are significantly greater in Stockholm, compared to national dispensings. Allergology is a specialty in Sweden, a country in which there are 70 Allergologists, corresponding to 0.71 Allergologists per 100,000 inhabitants (11). In 2023, half of allergy clinics in Sweden were located in Stockholm (34/67; 50.7%) (12), raising the possibility that access to allergy care and education may be greater in Stockholm than elsewhere in the country.

Epinephrine is the front-line medication for anaphylaxis (1). Yet, this life-saving medication remains unavailable (13), underprescribed and underused in many different countries, as evidenced by suboptimal dispensings, particularly amongst adults ages 19 + years and males (14). In the United States, where EAI are typically dispensed as twin-packs with out-of-pocket costs many times with a cost in excess of $600 USD (15), Warren et al. reported that 89% of patients do fill their EAI prescriptions (16). Amongst those who did not fill their EAI prescription, 25% reported cost barriers. The authors concluded that lower out-of-pocket EAI dispensings, coupled with patient education, may contribute to anaphylaxis management practices (16). While EAI are more costly in the USA than other countries (17), a recent review and meta-analysis demonstrate that, globally, pre-hospital use of epinephrine is suboptimal, albeit higher amongst children than adults, at 20.98% vs. 7.17%, respectively (18).

Yet, reduced or eliminated out-of-pocket expenses alone do not appear to correspond to increased EAI dispensings. In Japan, which has a universal health coverage system, out-of-pocket costs for EAI have been borne largely by the government since 2011, resulting in what has been described as having “zero or minimal cost” (19). While EAI dispensings to patients who experienced severe anaphylaxis in that country steadily and significantly increased from 2011 to 2016 (*p* < 0.003), a total proportion of only 28.8% of all patients were prescribed an EAI over the study period. Moreover, only 40.5% of patient refilled their EAI prescriptions annually (19). While the Swedish data used for the present study do not permit us to consider timing of dispensings or prescription refills, like the above-cited study from Japan (19), Swedish data do support that the absence of out-of-pocket expenses alone does not sustain increased EAI dispensings. We previously reported that, from 2006 to 2015, when modest out-of-pocket fees were attached to EAI dispensings, EAI were dispensed, on average, 7.74 EAI/1,000 males annually, compared to 6.01/1,000 females annually, then increased slightly for those ages 5–19 years during the two years immediately following the removal of out-of-pocket expenses. In this follow up study, dispensings were comparable to those reported in the original study, and which peaked in 2018, at 9.87/1,000 for males and 7.31/1,000 for females. Decreases were noted in the subsequent years. While it could be argued that these decreases may reflect widely-seen decreases in EAI dispensings during the COVID-19 pandemic, it warrants mentioned that Sweden was one of very few countries that did not have strict restrictions on public gatherings (20). To this end, a follow up study is needed in some years’ time to assess whether this decrease is a temporary artifact, or will be sustained over the longer term.

In Sweden, children ages 0–4 and 15–19 years were disproportionately, but not statistically significantly, more likely to be hospitalised than children from 5 to 14 years old. Recently, Robinson et al. from the United States reported statistically significant increases in ED visits for anaphylaxis between 2006 and 2015 across all age groups (0–3; 3–<6; 6–<12; and 12–<18) (21). In contrast to the Swedish findings however, the lowest number of visits in the United States were amongst the youngest age groups.

In 2019, the Swedish National Food Agency published revised guidelines that support the early introduction of common food allergens (22). A recent paper from the Swedish NorthPop Birth Cohort provides evidence that the proportion of families introducing common allergens, including egg and peanut, increased from 2016 to 2018 to 2019–2021 (23). The Swedish dataset used in the present study does not provide opportunity to examine the triggers of allergic reaction or narrower age groups than those presented herein. Nonetheless, it is interesting to note however, that, in Sweden, the number of annual inpatient stays were amongst those ages 0–4 years in the present update were similar to numbers were reported from 2006 to 2017 (5).

We acknowledge the limitations of our study, which was based on publicly available data. As such, we lacked detailed individual-level data that may have permitted analyses that adjusted for potentially confounding variables. We also lacked insight into the specific symptoms that contributed to individual-level coding for adverse reactions, not elsewhere classified.

Despite these limitations, this study is based on robust, national level data spanning six years. Consideration to age, sex and county gleaned insights into trends between groups amongst children. Further comparison with adult data provided evidence that trends differ between pediatrics and adults.

As indicated in our study and elsewhere (21), the greatest number of visits were reported amongst older children. Adolescents and young people are known to have suboptimal food allergy management behaviours (24, 25), which our findings support, persists even in the absence of EAI dispensed with zero out-of-pocket costs. Moreover, our findings reinforce the calls made by Khaleva et al. for specific training and standardisation of allergy management for healthcare professionals, to support adolescents as they transition to self-management of their condition (26).

Owing to the unique structure of the Swedish healthcare system, including the difference in out-of-pocket expenses for children vs. adults, comparisons of our findings with those from other countries are challenging. The healthcare systems most similar to that in Sweden are found in the countries neighbouring Sweden (27). To this end, comparisons with other Nordic countries are most appropriate. A recent Finnish study of allergy-related hospitalisations amongst adults provides evidence of an annual ∼1% between 1999 and 2000 (28). In the present study, we reported that, amongst children, hospitalisations for T78.0, adverse reactions, not elsewhere classified, fluctuated over the study period. Over a similar time period, from 2015 to 2020, pediatric hospitalisations for asthma, another allergic disease decreased in both Sweden and Finland (29). Reasons for a decrease in hospitalisations for one allergic disease (i.e., asthma) but others (i.e., adverse reactions, not elsewhere classified) are unclear, and warrant further exploration in a future study.

Finally, it warrants mention that the upward trend in hospitalisations for adverse reactions amongst those ages 0–4 years in 2019 and 2020 coincides with the publication of the Swedish guidelines for early introduction. Published in 2019, these guidelines promote the introduction of legumes, peanuts and tree nuts in the infant diet (30) and were widely communicated throughout the country (31). These foods, however, are common triggers of allergic reactions (32, 33).

In conclusion, in the sustained absence of out-of-pocket costs for EAI, dispensings remained stable across the study period, albeit with notable inter-county differences. More EAI were dispensed to children from age 5 years, compared to younger children. Allergy-related hospitalisations were highest amongst children ages 0–4, decreased amongst children ages 5–14 years, then increased amongst those ages 15–19 years.

## Data Availability

Publicly available datasets were analyzed in this study. This data can be found here: https://sdb.socialstyrelsen.se/if_lak/val.aspx.
