# Supplementary material for: Changes in epinephrine dispensings and allergy hospitalisations in Sweden in the years following the removal of autoinjector co-payments
Source: Front Allergy. 2024 Aug 16;5:1434461. doi: 10.3389/falgy.2024.1434461 (PMC11362073; doi:10.3389/falgy.2024.1434461)
Supplement: Supplementary file 1 [file Datasheet1.docx]

**Changes in epinephrine dispensings and allergy hospitalisations in Sweden in the years following the removal of autoinjector co-payments**

**______________________________________________________________________________________**

**Supplementary material**

**Table S1.** Dispensed epinephrine prescriptions per 1000 individuals,

from 2018 to 2023, in Sweden, stratified by children (ages 0-19 years)

and adults (ages 20-85+ years)…………………………………………………………………………………………………………2

**Figure S1.** Number of patients with annual EAI dispensings per 1000 individuals,

from 2018 and 2023, stratified by children (including by age group) and adults……………………………….4

**Table S1.** Dispensed epinephrine prescriptions per 1000 individuals, from 2018 to 2023, in Sweden, stratified by children (ages 0-19 years) and adults (ages 20-85+ years)

| **Region** | **Age group** | **2018** | **2019** | **2020** | **2021** | **2022** | **2023** |
| --- | --- | --- | --- | --- | --- | --- | --- |
| National | Children | **8.63*** | **8.26*** | **7.01*** | **6.65†** | **7.37*** | **6.70*** |
|  | Adults | **5.31** | **5.18** | **4.14** | **4.88** | **5.06** | **4.98** |
| Large rural areas |  |  |  |  |  |  |  |
| Dalarnas län | Children | 6.98 | 6.96 | 6.23 | 6.14 | 5.88 | 6.14 |
|  | Adults | 2.87 | 2.63 | 1.90 | 2.07 | 2.41 | 2.29 |
| Jämtlands län | Children | 4.34 | 3.41 | 3.19 | 3.14 | 3.07 | 3.07 |
|  | Adults | 4.92 | 4.92 | 3.72 | 4.72 | 4.68 | 4.38 |
| Norrbottens län | Children | 10.87 | 10.20 | 8.76 | 8.43 | 9.01 | 7.66 |
|  | Adults | 4.07 | 4.16 | 3.30 | 3.74 | 4.48 | 3.83 |
| Värmlands län | Children | 7.72 | 7.31 | 6.10 | 5.70 | 5.75 | 5.32 |
|  | Adults | 5.10 | 5.38 | 4.39 | 5.19 | 5.11 | 4.91 |
| Västerbottens län | Children | 11.34 | 9.84 | 8.73 | 9.24 | 8.97 | 8.16 |
|  | Adults | 3.90 | 3.78 | 3.16 | 3.47 | 3.76 | 3.39 |
| Västernorrlands län | Children | 9.28 | 8.86 | 6.95 | 7.24 | 7.80 | 7.01 |
|  | Adults | 4.58 | 4.03 | 3.37 | 4.19 | 3.74 | 3.86 |
| Other rural areas |  |  |  |  |  |  |  |
| Blekinge län | Children | 7.19 | 7.38 | 5.94 | 5.97 | 6.29 | 5.98 |
|  | Adults | 6.44 | 6.02 | 5.22 | 5.82 | 6.04 | 6.76 |
| Gävleborgs län | Children | 7.22 | 7.27 | 6.56 | 6.19 | 6.00 | 6.19 |
|  | Adults | 4.61 | 4.21 | 3.66 | 4.14 | 4.22 | 4.11 |
| Gotlands län | Children | 4.35 | 4.40 | 3.00 | 2.03 | 5.32 | 8.15 |
|  | Adults | 5.23 | 5.11 | 5.20 | 5.27 | 4.92 | 6.00 |
| Hallands län | Children | 10.68 | 9.38 | 7.57 | 7.68 | 8.11 | 6.95 |
|  | Adults | 5.42 | 5.42 | 3.82 | 5.20 | 5.34 | 4.97 |
| Jönköpings län | Children | 4.84 | 4.80 | 3.70 | 4.06 | 4.13 | 3.74 |
|  | Adults | 4.86 | 4.62 | 3.88 | 4.37 | 4.63 | 4.69 |
| Kalmar län | Children | 7.34 | 7.00 | 5.49 | 6.17 | 7.38 | 6.39 |
|  | Adults | 6.34 | 5.70 | 4.96 | 6.06 | 6.28 | 7.97 |
| Kronobergs län | Children | 5.09 | 4.33 | 4.02 | 4.22 | 4.29 | 4.01 |
|  | Adults | 4.79 | 4.69 | 3.31 | 4.37 | 4.48 | 4.88 |
| Norrbottens län | Children | 10.87 | 10.20 | 8.76 | 8.43 | 9.01 | 7.66 |
|  | Adults | 4.07 | 4.16 | 3.30 | 3.74 | 4.48 | 3.83 |
| Örebro län | Children | 4.88 | 4.99 | 4.21 | 4.31 | 4.72 | 4.16 |
|  | Adults | 4.70 | 4.21 | 4.34 | 4.18 | 4.64 | 4.56 |
| Östergötlands län | Children | 8.73 | 9.86 | **8.61*** | 7.48 | 9.21 | 8.88 |
|  | Adults | 6.14 | 5.48 | **4.66** | 5.57 | 5.77 | 6.49 |
| Södermanlands län | Children | 7.13 | 7.11 | 6.05 | 5.31 | 5.78 | 5.86 |
|  | Adults | 5.72 | 5.38 | 4.41 | 5.15 | 5.18 | 5.38 |
|  |  |  |  |  |  |  |  |
|  |  |  |  |  |  |  |  |
| Uppsala län | Children | 7.48 | 6.71 | 6.40 | 5.15 | 6.32 | 5.22 |
|  | Adults | 6.07 | 5.76 | 4.72 | 5.45 | 5.59 | 5.68 |
| Västmanlands län | Children | 4.56 | 4.54 | 3.78 | 3.13 | 3.85 | 3.47 |
|  | Adults | 4.54 | 4.37 | 3.16 | 3.99 | 4.09 | 3.77 |
| Three most populated counties | | | | | | | |
| Skåne län | Children | 6.59 | 6.23 | 5.55 | 4.96 | 5.68 | 5.03 |
|  | Adults | 5.25 | 5.06 | 3.87 | 4.89 | 4.95 | 4.91 |
| Stockholms län | Children | **12.46*** | **11.97*** | **10.06*** | **9.85†** | **11.06*** | **10.07*** |
|  | Adults | **5.94** | **6.02** | **4.69** | **5.40** | **5.65** | **5.40** |
| Västra Götalands län | Children | 7.90 | 7.31 | 6.10 | 5.41 | 5.88 | 5.08 |
|  | Adults | 5.14 | 5.11 | 4.07 | 4.82 | 4.97 | 4.62 |

*p-value<0.001 compared to national dispensings for corresponding age group and year

† p-value<0.01 compared to national dispensings for corresponding age group and year

**Figure S1.** Number of patients with annual EAI dispensings per 1000 individuals, from 2018 and 2023, stratified by children (including by age group) and adults
